# Longitudinal echocardiographic and clinical follow-up of patients undergoing mitral valve surgery without concomitant tricuspid valve repair

**DOI:** 10.1007/s12471-018-1159-4

**Published:** 2018-10-01

**Authors:** R. Jansen, B. R. van Klarenbosch, M. J. Cramer, R. C. A. Meijer, P. H. M. Westendorp, H. W. J. Meijburg, J. J. J. Bucx, S. A. J. Chamuleau, J. Kluin

**Affiliations:** 1 Department of Cardiology, University Medical Center Utrecht, Utrecht, The Netherlands; 2 Department of Cardiothoracic Surgery, University Medical Center Utrecht, Utrecht, The Netherlands; 3 Department of Cardiology, Beatrix Hospital Gorinchem, Gorinchem, The Netherlands; 4 Department of Cardiology, Jeroen Bosch Hospital ’s-Hertogenbosch, ’s-Hertogenbosch, The Netherlands; 5 Department of Cardiology, Diakonessenhuis Utrecht, Utrecht, The Netherlands; 6 Department of Cardiothoracic Surgery, Academic Medical Center Amsterdam, Amsterdam, The Netherlands

**Keywords:** Tricuspid valve repair, Functional tricuspid regurgitation, Mitral valve surgery, Mitral valve aetiology

## Abstract

**Background:**

In patients with mild to moderate functional tricuspid regurgitation (TR) and absence of right ventricular dysfunction or tricuspid annulus (TA) dilatation, there is currently no indication for concomitant tricuspid valve (TV) repair during elective mitral valve (MV) surgery. However, long-term results are conflicting. Here, we sought to determine the clinical outcome of this cohort, the rate of TR progression after MV surgery and the role of MV aetiology.

**Methods:**

Patients for elective MV surgery without concomitant TV repair were retrospectively analysed with longitudinal echocardiographic and clinical follow-up, focusing on TR progression and MV aetiology. Linear regression analysis was performed for change in TR at follow-up, using pre-determined variables and confounders.

**Results:**

In total 204 patients without TV repair were analysed. Development of more than moderate TR after a median of 3.1 [1.6–4.6] years was rarely seen: only in 2 out of 161 patients (1.2%) with known TR grade at follow-up. Overall, median preoperative and late postoperative TR grade were equal (*p* = 0.116). Subanalysis showed no significant difference in MV aetiology subgroups. Preoperative TR grade and male gender were inversely correlated to change in TR. Mortality was not influenced by the 1‑year postoperative TR severity.

**Conclusion:**

Our data showed that in a study population of patients with mild to moderate TR undergoing MV surgery without concomitant TV repair, significant late TR was rarely seen. Based on our study, it is safe to waive concomitant TV repair in this specific patient cohort.

**Electronic supplementary material:**

The online version of this article (10.1007/s12471-018-1159-4) contains supplementary material, which is available to authorized users.

## What’s new

- Guidelines recommend concomitant tricuspid valve (TV) repair for severe tricuspid regurgitation (TR); however, less is known regarding the management of mild to moderate TR.
- Our study showed that in patients with moderate TR or less, undergoing mitral valve (MV) surgery without concomitant TV repair, significant late functional TR was seldom seen, and change in TR severity was not influenced by the MV aetiology.
- Clinical decision-making regarding concomitant TV repair during MV surgery can be safely based on the preoperative evaluation of TR grade: it is safe to waive concomitant TV repair in our specific patient cohort.

## Introduction

Mitral valve (MV) disease represents an increasing health burden, due to ageing and population growth [1]. Approximately 30–50% of patients with severe mitral regurgitation (MR) have significant tricuspid regurgitation (TR) [2]. Functional TR carries an adverse prognosis which is related to its severity [3]. It was historically believed that TR may improve after correction of the MV pathology [4]. However, recent data have shown an increase in TR in a still unclassified subgroup, irrespective of residual or recurrence of MV disease or preoperative TR [5–8]. Reoperation may be associated with high mortality [9]. Therefore, guidelines recommend concomitant tricuspid valve (TV) repair for severe TR (class I), as it improves mortality and morbidity in these patients [10–12]. However, less is known regarding the management of mild to moderate TR. Recent guidelines recommend concomitant tricuspid annuloplasty for a tricuspid annulus (TA) diameter of ≥40 mm or >21 mm/m^2^ (class IIa) regardless of the TR severity, solely based on expert opinion [10, 11, 13–15]. While concomitant TV repair has proven to be a safe procedure [14, 16–18], it seems severely underutilised in daily practice [12, 18–20]. Insight into the longitudinal echocardiographic and clinical follow-up, including identification of risk factors for TR progression, is of importance to evaluate and complement current guidelines.

The purpose of this study was to evaluate the echocardiographic and clinical results in patients with moderate TR or less, undergoing MV surgery without concomitant TV repair, in order to: (1) analyse postoperative TR progression and clinical outcome, and (2) evaluate the role of MV aetiology as potential risk factor for postoperative TR progression.

## Methods

### Study population

**Fig. 1:**
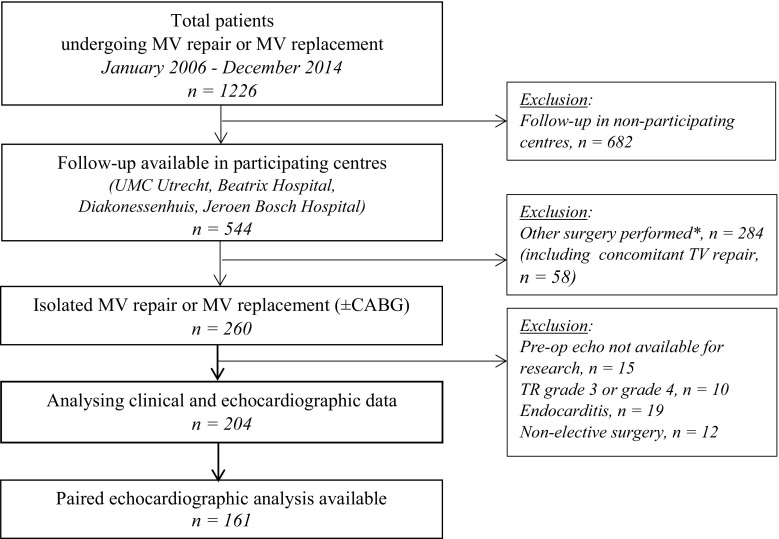
Study flowchart (*MV* mitral valve, *UMC* University Medical Center, *TV* tricuspid valve, *CABG* coronary artery bypass grafting, *TR* tricuspid regurgitation, *TTE* transthoracic echocardiography, * excluding concomitant TV repair)

Between 2006 and 2014 a total of 1,226 patients underwent MV surgery in the University Medical Center Utrecht (UMCU), the Netherlands (Fig. 1). We analysed the two-dimensional (2D) transthoracic echocardiograms (TTE) and clinical data of 204 patients meeting the inclusion criteria: (1) age ≥18 years; (2) preoperative TR grade <3; (3) referred for elective MV surgery with or without coronary artery bypass grafting and no concomitant TV repair or other concomitant procedures (e. g. MAZE or aortic valve surgery); and (4) follow-up in a participating centre. Our study was approved by the institutional review board of the UMCU, which waived patient consent.

### Data collection

Preoperative and perioperative information was retrieved from the surgical database of the UMCU. Postoperative follow-up data and echocardiographic images and/or reports were obtained from the treating physician. Re-evaluation of the images was performed off-line using Xcelera software (Philips Healthcare, the Netherlands). The routine evaluation of echocardiograms was performed by experienced sonographers. Echocardiographic measurements were obtained in accordance with the guidelines [21, 22]. Left ventricular (LV) and right ventricular (RV) function, and atrial and ventricular dimensions were qualified and, if possible, quantified. The severity of valvular disease was graded 0–4 (including grade 0.5 for trace severity) by a cardiologist of the participating centre with a special interest in cardiac imaging, using an integrative approach based on the echocardiographic criteria as recommended by the European guidelines [10, 21]. For data analysis TR grade was divided into four groups: none, trace or mild, moderate, and more than moderate TR. Additionally the change in TR grade between the preoperative and most recent TTE was determined. In case of insufficient data regarding TR grade and TA dimensions, the images were re-evaluated in the apical 4‑chamber view. TA was considered dilated when ≥40 mm [21]. Each patient was allocated to either the *organic *or *functional *MV subgroup: organic when a specific component of the MV apparatus was diseased, or functional when caused by secondary changes induced by abnormal ventricular size and leaflet retraction.

### Statistical analysis

Statistical analysis was done using SPSS (version 21.0, IBM Corporation, New York). Continuous variables were expressed as mean (±SD) and compared using Student’s t‑test in case of normally distributed data, or expressed as median (interquartile range) and compared using Wilcoxon signed-rank test or Mann-Whitney U test for non-normal distribution. One-way ANOVA or Kruskal-Wallis was used to compare >2 unpaired groups. Categorical data were described using frequencies and percentages. We performed comparative evaluations via the χ^2^ or McNemar’s test for binary results, and χ^2^ or Wilcoxon signed-rank test in case of ordinal data. The Kaplan-Meier method was used to calculate long-term survival for the different grades of 1‑year postoperative TR. Statistical significances between the survival curves was determined by a log-rank test; the *p*-value <0.05 was considered statistically significant. Ordinal regression analysis for change in TR grade (between preoperative and most recent TTE) was performed based on complete case analysis, including univariable and multivariable ordinal regression on pre-determined variables of interest at baseline (MR grade, MV aetiology, MV aetiology subcategories, TR grade, TA diameter, RV function, RA dilatation, LV function, LA dilatation and gender) and potential confounders (age, NYHA class, pacemaker implantation and atrial fibrillation).

## Results

### Patient characteristics

Baseline characteristics are shown in Table 1. In Online Resource 1 (Supplementary material) the subcategories per MV aetiology are depicted. Patients with functional MV disease (30.4%) were significantly older compared with the organic subgroup (69.6%). Also NYHA class, EuroSCORE and rates of the comorbidities hypertension, chronic obstructive pulmonary disease, known coronary artery disease, diabetes, and renal failure were significantly higher in the functional aetiology subgroup, particularly for ischaemic MR. Preoperative MV annulus dilation was present in 58.5% of patients with organic and >90% of patients with functional valve disease. Clinically important mitral stenosis (MS) was only seen in the organic subgroup (9.9%). MV repair was more often performed in patients with functional disease (96.8%), compared with 18.3% MV replacement in the organic aetiology subgroup (mainly for rheumatic disease or severe calcifications). Patients with more preoperative TR showed a higher NYHA class (*p* = 0.011). Regarding the surgical characteristics, more MV replacements were performed in patients with moderate TR at baseline compared with lower grades (*p* = 0.017). Standard median sternotomy was performed in 98.5%. Three patients underwent a right anterior thoracotomy. Access to the MV was achieved through a left atriotomy.

**Table 1:** Baseline characteristics and procedure details (*n* = 204)

*Patient Characteristics*	
Age (years)	61.1 ± 13.1
Male gender	125 (61.3)
BSA (m^2^)	2.2 ± 0.2
NYHA class ≥III	92 (45.1)
EuroSCORE II	1.4 [0.8–3.5]
Hypertension	63 (30.9)
Known coronary artery disease (*n* = 203)	84 (40.9)
Diabetes	23 (11.3)
COPD	18 (8.8)
Renal failure	38 (18.6)
Atrial fibrillation	38 (18.6)
Pacemaker or ICD	8 (3.9)
*Surgical Characteristics*	
Organic MV disease	142 (69.6)
– Myxomatous degeneration	79 (38.7)
– Fibroelastic degeneration	47 (23.0)
– Rheumatic disease	13 (6.4)
– Other	3 (1.5)
Functional MV disease	62 (30.4)
– Ischaemic cardiomyopathy	46 (22.5)
– Other	16 (7.8)
Preoperative MR (*n* = 203)	
>Moderate	164 (80.2)
Preoperative TR	
– No	29 (14.2)
– Trace or mild	126 (61.8)
– Moderate	49 (24.0)
>Moderate	0 (0.0)
Preoperative TA ≥ 40 mm (*n* = 165)	14 (8.5)
Preoperative TA diameter (mm) (*n* = 165)	33 ± 4
MV replacement	28 (13.7)
– Mechanical	17 (8.3)
– Bioprosthesis	11 (5.4)
MV repair	176 (86.3)
– Physio ring	137 (67.2)
– Cosgrove band	38 (18.6)
Elevated RVSP (*n* = 118)	51 (43.2)
Concomitant CABG	75 (36.8)
Cross-clamp time (minutes)	118 [92–149]

Data are depicted as *n* (%), mean ± SD or median [interquartile range]

*BSA* body surface area, *NYHA* New York Heart Association, *COPD* chronic obstructive pulmonary disease, *ICD* implantable cardioverter-defibrillator, *MV* mitral valve, *MR* mitral regurgitation, *TR* tricuspid regurgitation, *TA* tricuspid annulus, *RVSP* right ventricular systolic pressure, *CABG* coronary artery bypass crafting

### Clinical results

**Fig. 2:**
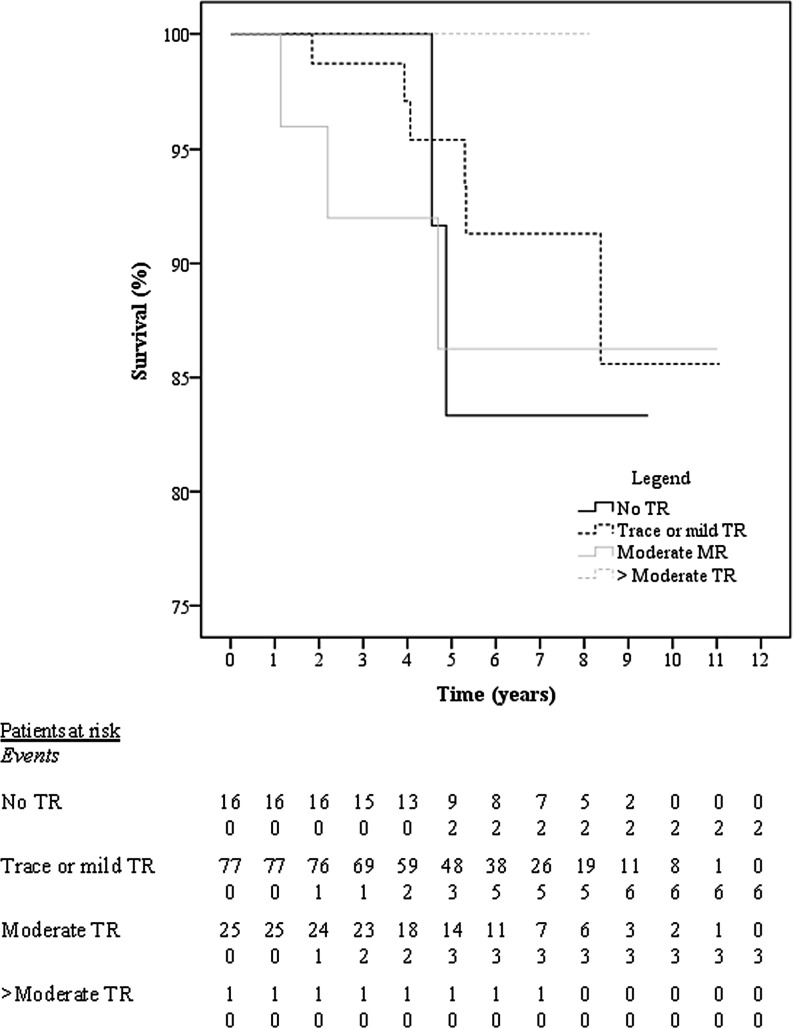
Kaplan-Meier to illustrate survival according to grade of tricuspid regurgitation at 1 year postoperatively (*p* = 0.972). Number at risk and number of events are depicted

Clinical outcomes are depicted in Table 2. Of the 204 patients, 5 (2.5%) died within 30 days (shock *n* = 3, respiratory failure *n* = 1, acute neoplastic disease *n* = 1). All had ischaemic MR. Reoperation <30 days after surgery occurred in 9.3%, mainly for bleeding complications (7.8%). Of the patients with postoperative AF, this rhythm was already present at baseline in 29.8%. The prevalence of AF at follow-up was similar to baseline, whereas the NYHA class ≥III significantly decreased. The overall survival after a median follow-up of 5.5 [3.7–8.1] years was 87.3%, and significantly better for the organic compared with functional MV aetiology (92.3% versus 75.8%). The highest mortality rate was seen for ischaemic MR (28.3%). There was no significant difference in survival according to TR grade at 1 year (Fig. 2, *p* = 0.972). In 50% of the deceased patients TR was never severe and therefore not a cause of mortality; the TR grade was unknown in the remaining subjects.

**Table 2:** Clinical results at follow-up and echocardiographic characteristic at most recent follow-up

*Clinical Characteristics*	
*Early outcome*	
Operative mortality	0 (0.0)
30 days mortality	5 (2.5)
New permanent pacemaker or ICD	5 (2.5)
Atrial fibrillation	104 (51)
Reoperation	19 (9.3)
– due to bleeding	16 (7.8)
– other	3 (1.5)
*Late outcome*	
Postoperative (for most recent FU analysis) (years)	3.4 [1.8–5.2]
Postoperative (for survival analysis) (years)	5.5 [3.7–8.1]
Mortality	26 (12.7)
– Cardiovascular	20 (9.8)
– Other	6 (2.9)
NYHA class ≥III (*n* = 184)	12 (6.5)
New reoperation (*n* = 184)	7 (3.8)
– Due to mitral stenosis post MV surgery	1 (0.5)
– Due to endocarditis	2 (1.0)
– Due to bleeding	1 (0.5)
Rehospitalisation (*n* = 187)	66 (35.3)
– Cardiovascular	46 (22.5)
Congestive heart failure (*n* = 183)	19 (10.4)
New permanent pacemaker or ICD (*n* = 185)	14 (7.6)
Atrial fibrillation (*n* = 183)	38 (20.8)
OAC (*n* = 180)	78 (43.3)
Stroke (*n* = 191)	2 (1.0)
*Echocardiographic Characteristics*	
Time after surgery, years (*n* = 164)	3.1 [1.6–4.6]
LV function, *n* (%) (*n* = 160)	
– Poor (EF < 30%) or moderate (EF 30–44%)	33 (20.6)
LV EF, % ± SD (*n* = 103)	55 ± 15
LA dilatation, *n* (%) (*n* = 135)	
– Moderate or severe	34 (25.2)
RA dilatation, *n* (%) (*n* = 155)	
– Moderate or severe	11 (7.1)
RV function, *n* (%) (*n* = 147)	
– Poor or moderate	11 (7.5)
TAPSE, cm ± SD (*n* = 129)	1.8 ± 0.5
MR grade, *n* (%) (*n* = 159)	
>Moderate	3 (1.9)
TR grade, *n* (%) (*n* = 161)	
– No	24 (14.9)
– Trace or mild	107 (66.5)
– Moderate	28 (17.4)
>Moderate	2 (1.2)
TA ≥ 40 mm, *n* (%) (*n* = 154)	23 (14.9)
– Of whom had pre-operative TA ≥ 40 mm	3 (13.0)
Elevated RVSP, *n* (%) (*n* = 97)	14 (14.4)

Data are depicted as *n* (%), mean ± SD or median [interquartile range]

*ICD* implantable cardioverter-defibrillator, *NYHA* New York Heart Association, *MR* mitral regurgitation, *OAC* oral anticoagulation, *LV* left ventricular, *EF* ejection fraction, *LA* left atrial, *RA* right atrial, *TAPSE* tricuspid annular plane systolic excursion, *TR* tricuspid regurgitation, *TA* tricuspid annulus, *RVSP* right ventricular systolic pressure.

### Echocardiographic results

**Fig. 3:**
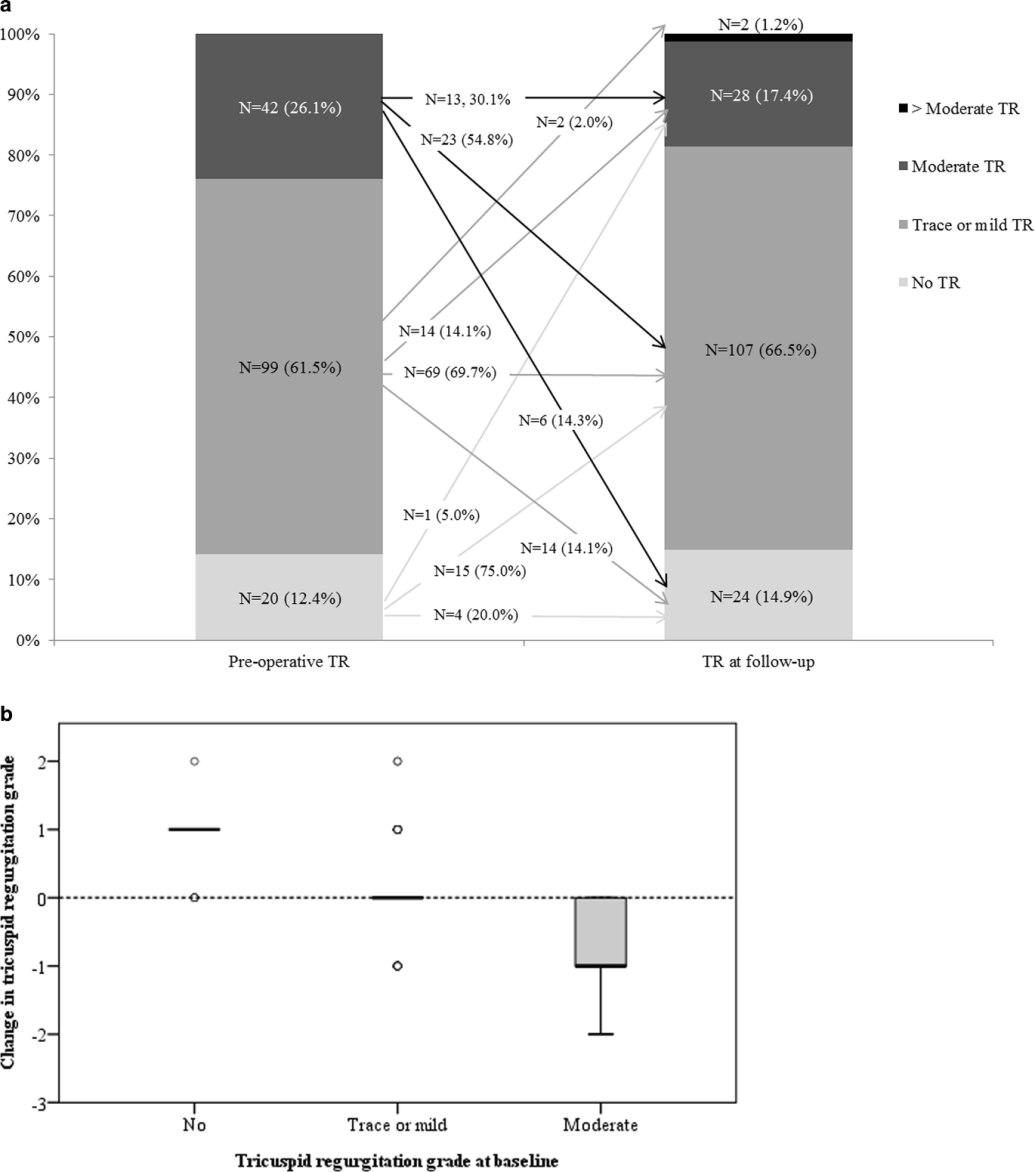
Grade of tricuspid regurgitation (**a**), and change in severity per grade at baseline (**b**) in patients with known tricuspid regurgitation severity at both baseline and most recent follow-up echocardiogram (*n* = 161, *TR* tricuspid regurgitation). **a** Median (IQR) tricuspid regurgitation grade at baseline: 1.0 (1.0–2.0), and most recent follow-up: 1.0 (1.0–1.0), *p* = 0.166. **b** Median (IQR) change in patients with no tricuspid regurgitation at baseline: 1.0 (1.0–1.0), mild or trace tricuspid regurgitation at baseline: 0.0 (0.0–0.0), and moderate tricuspid regurgitation at baseline: −1.0 (−1.0– −1.0), *p* < 0.0001

**Fig. 4:**
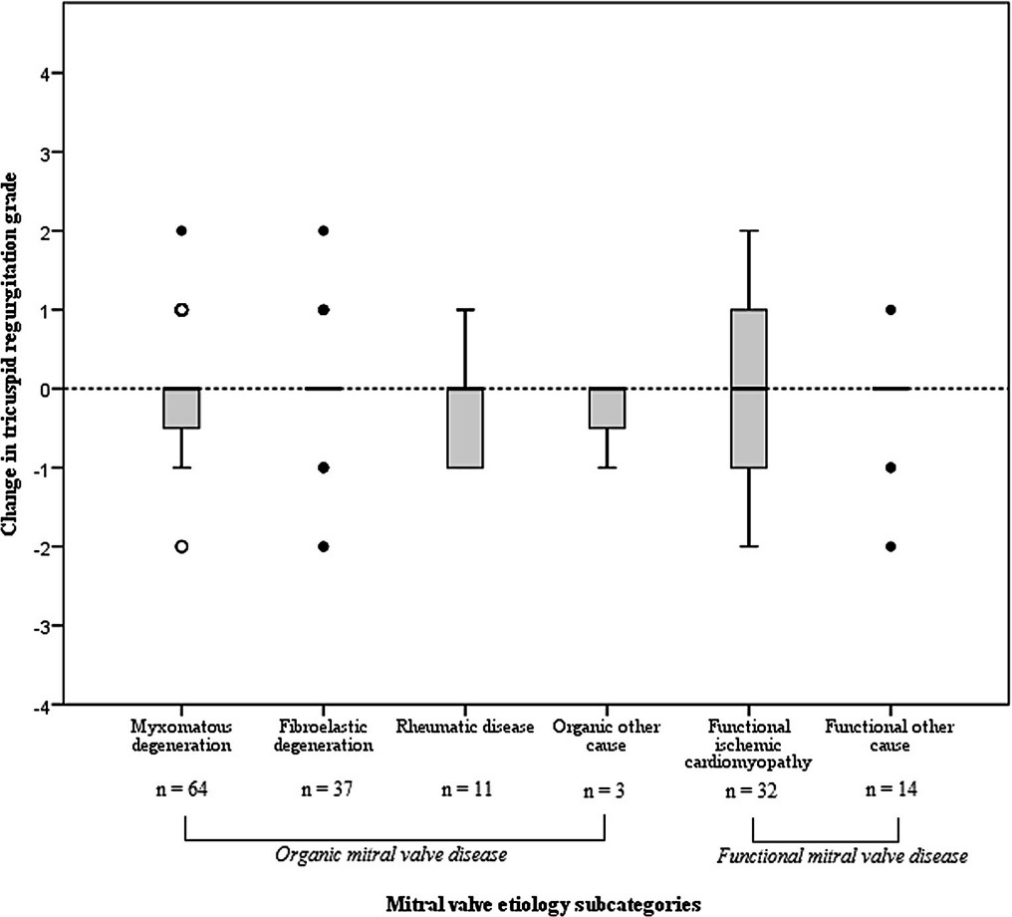
Change in grade of tricuspid regurgitation per mitral valve aetiology subcategory (Median (IQR) change in myxomatous degeneration: 0.0 (−0.8–0.0), fibroelastic degeneration: 0.0 (0.0–0.0), rheumatic disease: 0.0 (−1.0–0.0), organic other cause: 0.0 (−1.0–NA), functional ischaemic cardiomyopathy: 0.0 (−1.0–1.0), functional other cause: 0.0 (−0.3–0.0), *p* = 0.575)

Overall echocardiographic results at follow-up are shown in Table 2. In 204 patients echocardiographic data were available at baseline. Postoperatively, the TTE was available in 202 patients (2 subjects passed away), while TR grade could be determined in 161 patients using the echocardiographic data or images (78.9%). Fig. 3 shows the overall change, including median changes per baseline TR grade subgroups. In general, almost all patients with no TR at baseline developed some TR at late follow-up, whereas in subjects with preoperatively moderate TR an increase was never seen. Overall change in TR severity was limited and not significant: in only 3 out of 161 patients (1.9%) was a change in TR severity of >1 grade seen, whereas TR severity did not change in 86 patients (53.4%). RV systolic function as measured by tricuspid annular plane systolic excursion (TAPSE) decreased from 2.4 ± 0.5 cm preoperatively to 1.8 ± 0.5 cm (*p* < 0.001) at late follow-up. Medians of TR grade were similar amongst the MV aetiology groups (Table 3). Long-term follow-up showed 2 patients with TR grade ≥3 (Fig. 4); one in each of the MV aetiology subgroups. Median changes in TR grade were never significant over time in either the MV aetiology subgroup or the MV aetiology subcategories (Fig. 4). Baseline MR severity was significantly lower in the functional compared with the organic group (median 3.0 versus 4.0 respectively). Both preoperative and postoperative LV and RV function were poorer in functional MV disease.

**Table 3:** Echocardiographic results in patients with organic and functional mitral valve disease

	Organic (*n* = 142)	Functional (*n* = 62)	*P*-value
*Baseline*			
LV function (*n* = 141/61)			
– Poor (EF < 30%) or moderate (EF 30–44%)	4 (2.8)	25 (41.0)	<0.0001
LA dilatation (*n* = 131/55)			
– Moderate or severe	75 (57.3)	27 (49.1)	0.060
RA dilatation (*n* = 132/54)			
– Moderate or severe	14 (10.6)	6 (11.1)	0.940
RV function (*n* = 127/55)			
– Poor or moderate	1 (0.8)	3 (5.5)	0.003
TAPSE (cm) (*n* = 87/43)	2.3 [2.1–2.8]	2.0 [1.8–2.5]	0.001
MR grade (*n* = 141/62)			
>Moderate	122 (86.5)	42 (67.7)	<0.0001
TR grade (*n* = 142/62)			0.892
– No	21 (14.8)	8 (12.9)	
– Trace or mild	88 (62.0)	38 (61.3)	
– Moderate	33 (23.2)	16 (25.8)	
>Moderate	0 (0.0)	0 (0.0)	
TA ≥ 40 mm (*n* = 115/50)	8 (7.0)	6 (12.0)	0.285
TA diameter (mm) (*n* = 115/50)	33 ± 4	33 ± 5	0.994
*MOST RECENT FOLLOW-UP*			
LV function (*n* = 114/46)			
– Poor (EF < 30%) or moderate (EF 30–44%)	10 (8.3)	23 (50.0)	<0.0001
LA dilatation (*n* = 94/41)			
– Moderate or severe	23 (24.5)	11 (26.8)	0.181
RA dilatation (*n* = 109/46)			
– moderate or severe	7 (6.4)	4 (8.7)	0.596
RV function (*n* = 105/42)			
– Poor or moderate	3 (2.9)	8 (19.0)	0.002
TAPSE (cm) (*n* = 90/39)	1.9 [1.6–2.1]	1.6 [1.4–2.1]	0.021
MR grade (*n* = 114/45)			
>Moderate	2 (1.8)	1 (2.2)	0.218
TR grade (*n* = 115/46)			0.754
– No	16 (13.9)	8 (17.4)	
– Trace or mild	79 (68. 8)	28 (60.9)	
– Moderate	19 (16.5)	9 (19.6)	
>Moderate	1 (0.9)	1 (2.2)	
TA ≥ 40 mm (*n* = 111/43)	18 (16.2)	5 (11.6)	0.617
TA diameter (mm) (*n* = 110/43)	34 ± 5	34 ± 5	0.869
Change in TR grade (*n* = 115/46)			0.569
−2	4 (3.5)	2 (4.3)	
−1	23 (20.0)	14 (30.4)	
0	66 (57.4)	20 (43.5)	
1	20 (17.4)	9 (19.6)	
2	2 (1.7)	1 (2.2)	

Data are depicted as *n* (%), mean ± SD or median [interquartile range]

*EF* ejection fraction, *LV* left ventricular, *LA* left atrial, *RA* right atrial, *RV* right ventricular, *TAPSE* tricuspid annular plane systolic excursion, *MR* mitral regurgitation, *TR* tricuspid regurgitation, *TA* tricuspid annulus, *RVSP* right ventricular systolic pressure.

### Regression analysis

Univariable regression analysis (Online Resource 2, supplementary material) showed a significant, negative correlation between change in TR grade and TR severity, TA diameter at baseline, and LA dilatation (regression coefficient (r) = −2.774, *p* < 0.0001, *r* = −0.839, *p* = 0.021, and *r* = −0.276, *p* = 0.024 respectively). In a multivariable regression model correcting for other parameters and confounders, only baseline TR grade and male gender remained independently correlated (*r* = −2.908, *p* < 0.0001 and *r* = −0.710, *p* = 0.027 respectively), suggesting more improvement of TR in patients with higher TR grade preoperatively, and more improvement in males compared with females.

## Discussion

Our study of patients with moderate TR or less, undergoing MV surgery without concomitant TV repair, revealed three important findings. First, more than moderate late functional TR was seldom seen in this cohort at a median follow-up of 3.1 years. Secondly, overall change in TR severity was not significant when comparing the preoperative and late postoperative TR grade. Lastly, change in TR severity was not influenced by MV aetiology. Therefore our data suggest that clinical decision-making regarding concomitant TV repair during MV surgery in patients with moderate TR or less, can be safely based on the preoperative evaluation of TR grade.

### Prevalence and change of TR

Several factors contribute to TR in MV disease. First, an increase in LA pressure may result in pulmonary hypertension and subsequent RV enlargement, remodelling and dysfunction. Consequently, the TA diameter increases, leading to leaflet tethering and/or papillary muscle displacement [23]. Second, MV disease may induce AF resulting in TR. Lastly, the TV may be affected by the same disease process as the MV aetiology leading to MR. In our study, only 2 (1.2%) patients developed more than moderate TR, in contrast with previous data showing a late significant TR prevalence ranging from 8–74% [6, 8, 14, 24–26]. A possible explanation for the non-significant change and therefore low prevalence of TR grade ≥3 in our cohort is the frequent performance of concomitant TV repair in patients with a TA annulus ≥40 mm. A proactive acquittal of the guidelines may have led to a selection bias, including less subjects with a larger TA diameter. Indeed, a brief evaluation of the TA diameter in patients: (1) without concomitant TV repair and TR grade <3 (current study cohort), (2) with concomitant TV repair and TR grade <3, and (3) with concomitant TV repair and TR grade ≥3, revealed a mean of 33 ± 4 mm, 42 ± 4 mm and 46 ± 6 mm respectively. Still, in 14 of the 165 patients with an available TA diameter (8.5%), the annulus at baseline was dilated, of whom 3 also showed a dilated TA at follow-up. Additionally, differences may depend on heterogeneity in study populations and outcome definitions. Also the follow-up period to determine significant TR remains controversial. Goldstone et al. showed that late postoperative TR is a slow progressive disease, with a marked increase (grade ≥3) after 9 years [8]. Our shorter follow-up period may therefore have led to a premature non-significant change in TR, possibly reflected by the decrease in RV systolic function (based on TAPSE) in our study population. However, other studies revealed a prevalence of significant TR (grade >2) in 48.6% and 18.2% after just 2 years [27, 28]. Lastly, our data do confirm guideline recommendations to defer concomitant TV repair in patients with non-severe TR: moderate TR at baseline decreased (69%) or remained unchanged (31%) in all subjects, and therefore TR never worsened. Although previous studies confirm these results [29], others have revealed TR grade >2 as being a risk factor for TR progression [7], possibly resulting from structural remodelling of, for example, RV and TA in patients with cardiomyopathy-related MV disease [30]. However, we expect less influence of structural remodelling on TR progression in our patient cohort, while 69.6% suffered from organic MV disease. In line with the idea of TR grade >2 being a risk factor, Chikwe et al. showed a higher 7‑year freedom from moderate or worse TR after concomitant TV repair compared with solely MV surgery in patients with moderate severity at baseline. On the contrary, the risk of developing moderate TR in subjects with no or mild regurgitation at baseline was low (17% versus 15%), matching our data. Nevertheless, their follow-up period was long (7 versus 3.1 years) [26]. Remarkably, our data more frequently showed a progression of TR in patients with lower baseline TR grade (probably explained by the regression toward the mean phenomenon), and female subjects [24, 29], although this was seldom significant TR and did not lead to excess mortality.

### MV aetiology as a potential risk factor

Several studies have reported a high prevalence of postoperative TR in rheumatic MV [20, 24] and functional MR, with rheumatic MR being reported as an independent risk factor for late significant TR [20, 24, 25, 27, 31]. Our study showed TR progression towards more than moderate in only 2 patients (1.2%). There was no significant correlation between increased TR grade and MV aetiology, but this subanalysis was not very powerful. Differences at baseline and follow-up between the MV aetiology subgroups (e. g. lower survival and worse LV and RV function) can be explained by the poorer outcome in patients with ischaemic cardiac disease.

### Study limitations

This study was retrospective with its inherent limitations. Only 17% of the 1,226 patients undergoing MV surgery in the UMC Utrecht were included. Main reasons are the exclusion of patients with follow-up in non-participating centres (682), and exclusion of patients with concomitant surgery (284). In addition, evaluation of 2D TTE in daily practice is limited by a poor imaging window, and especially TR-related measurements could not always be adequately obtained, further reducing the number of patients for clinical and echocardiographic data analysis towards 204, and for paired echocardiographic evaluation towards 161 subjects. Also standardised TR grading could not be carried out. Moreover, the surgical procedures performed in a single academic centre may not be applicable to other centres. Due to a low prevalence of late TR grade ≥3 postoperatively, no analyses for this endpoint could be performed, and no risk factors for significant postoperative TR were defined. A prospective study with longer follow-up is recommended to confirm our results.

## Conclusion

In patients with mild to moderate TR who underwent elective MV surgery without concomitant TV repair, our study showed that significant late functional TR was seldom seen. Change in TR severity in the late postoperative period was not influenced by the MV aetiology, and mortality was not correlated to 1‑year postoperative TR severity. According to our study, it is safe to waive concomitant TV repair in this specific patient cohort, which is relevant for clinical decision-making in the heart team.

## Caption Electronic Supplementary Material

Online Resource 1: Subcategories for the types of mitral valve etiology (figure). Online Resource 2 reflects the univariable and multivariable analysis in change in tricuspid regurgitation grade (*n* = 161) between baseline and most recent echocardiographic follow-up, and pre-determined variables of interest at baseline (table)
