# Supplementary material for: Longitudinal echocardiographic and clinical follow-up of patients undergoing mitral valve surgery without concomitant tricuspid valve repair
Source: Neth Heart J. 2018 Oct 1;26(11):552–61. doi: 10.1007/s12471-018-1159-4 (PMC6220025; doi:10.1007/s12471-018-1159-4)
Supplement: Supplementary file 1 — Online Resource 1: Subcategories for the types of mitral valve etiology (figure). Online Resource 2 reflects the univariable and multivariable analysis in change in tricuspid regurgitation grade (n = 161) between baseline and most recent echocardiographic follow-up, and pre-determined variables of interest at baseline (table) [file 12471_2018_1159_MOESM1_ESM.docx]

**Supplementary Material**

*Article title*: Longitudinal echocardiographic and clinical follow-up of patients undergoing mitral valve surgery without concomitant tricuspid valve repair

*Journal name*: Netherlands Heart Journal

*Author names*: R. Jansen, B.R. van Klarenbosch, M.J. Cramer, R.C.A. Meijer, P.H.M. Westendorp, H.W.J. Meijburg, J.J.J. Bucx, S.A.J. Chamuleau, J. Kluin

*Contact details*

*corresponding author*: Prof dr. S.A.J. Chamuleau, department of Cardiology University Medical Center Utrecht. Heidelberglaan 100, 3584 CX UTRECHT
E-mail: s.a.j.chamuleau@umcutrecht.nl; Tel. +31 88 75 67903

This document includes Online Resource 1 and 2.

ONLINE RESOURCE 1.

**Results**

Figure 1. Subcategories for the types of mitral valve etiology: a) organic (n=142), and b) functional (n=62)

a.


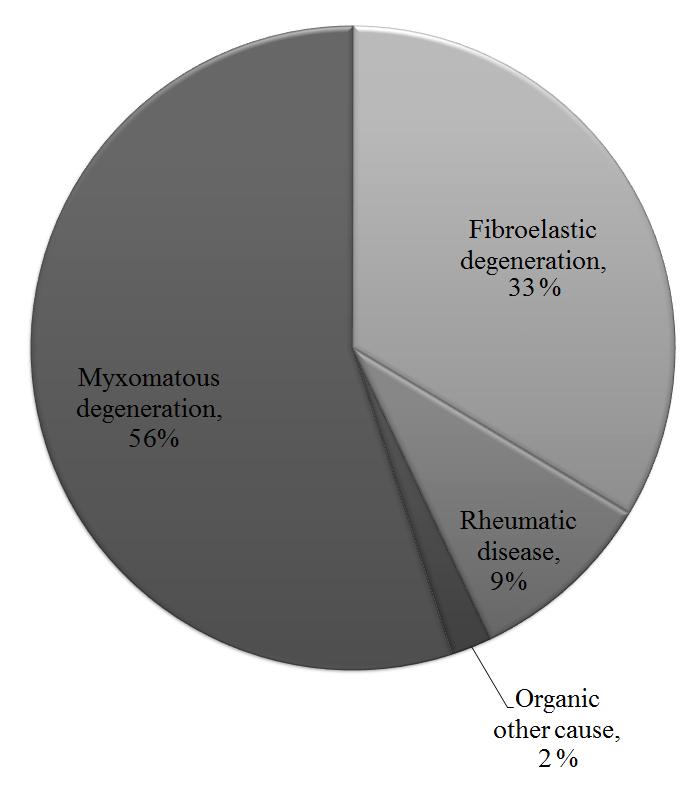


b.


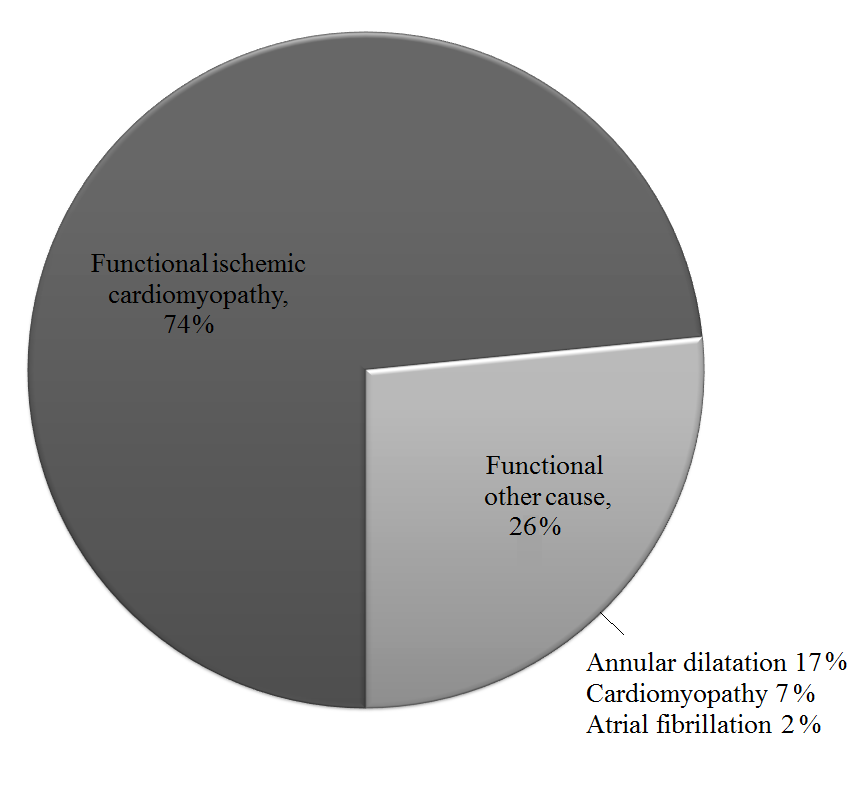


ONLINE RESOURCE 2.

**Results**

Table 1. Univariable and multivariable analysis in *change in tricuspid regurgitation grade* (n=161) between baseline and most recent echocardiographic follow-up, and pre-determined variables of interest at baseline

|  | **Univariable analysis** |  | **Multivariable analysis*** |  |
| --- | --- | --- | --- | --- |
| *Variables at baseline* | *Regression coefficient* | *P-value* | *Regression coefficient* | *P-value* |
| Mitral regurgitation grade (n=160) | 0.169 | 0.356 | 0.147 | 0.438 |
| Organic mitral valve etiology (n=161) | 0.259 | 0.435 | 0.189 | 0.588 |
| Mitral valve etiology subcategories (n=161) | -0.390 | 0.515 | 0.252 | 0.678 |
| Tricuspid regurgitation grade (n=161) | -2.774 | *0.000* | -2.908 | ***0.000*** |
| Tricuspid annulus diameter (n=135) | -0.839 | *0.021* | -0.768 | 0.301 |
| Right ventricular function (n=148) | 0.708 | 0.089 | 0.449 | 0.305 |
| Right atrial dilatation (n=150) | -0.302 | 0.159 | -0.414 | 0.153 |
| Left ventricular function (n=159) | 0.169 | 0.249 | 0.114 | 0.520 |
| Left atrial dilatation (n=149) | -0.276 | *0.024* | -0.246 | 0.052 |
| Male gender (n=161) | -0.483 | 0.118 | -0.710 | ***0.027*** |
| ** Based on n=118*  *Corrected for: age, New York Heart Association class, pacemaker implantation, atrial fibrillation* | | | | |
